# A Natural Antimicrobial Agent: Analysis of Antibacterial Effect and Mechanism of Compound Phenolic Acid on *Escherichia coli* Based on Tandem Mass Tag Proteomics

**DOI:** 10.3389/fmicb.2021.738896

**Published:** 2021-11-29

**Authors:** Geyin Zhang, Yunqiao Yang, Fareed Uddin Memon, Kaiyuan Hao, Baichang Xu, Shuaiyang Wang, Ying Wang, Enyun Wu, Xiaogang Chen, Wenguang Xiong, Hongbin Si

**Affiliations:** ^1^College of Animal Science and Technology, Guangxi University, Nanning, China; ^2^College of Veterinary Medicine, South China Agricultural University, Guangzhou, China

**Keywords:** compound phenolic acids, *Escherichia coli*, TMT, antibacterial mechanism, new antimicrobial agent

## Abstract

The objective of this study was to evaluate the antibacterial mechanisms of phenolic acids as natural approaches against multi-drug resistant *Escherichia coli* (*E. coli*). For that purpose, five phenolic acids were combined with each other and 31 combinations were obtained in total. To select the most potent and effective combination, all of the obtained combinations were examined for minimum inhibitory concentration (MIC) and it was found that the compound phenolic acid (CPA) 19 (protocatechuic acid, hydrocinnamic acid, and chlorogenic acid at concentrations of 0.833, 0.208, and 1.677 mg/mL, respectively) showed better efficacy against *E. coli* compared to other combinations. Furthermore, based on tandem mass tag (TMT) proteomics, the treatment of CPA 19 significantly downregulated the proteins associated with resistance (Tsr, Tar, CheA, and CheW), OmpF, and FliC of multidrug-resistant *E. coli*. At the same time, we proved that CPA 19 improves the sensitivity of *E. coli* to antibiotics (ceftriaxone sodium, amoxicillin, fosfomycin, sulfamonomethoxine, gatifloxacin, lincomycin, florfenicol, cefotaxime sodium, and rifampicin), causes the flagellum to fall off, breaks the structure of the cell wall and cell membrane, and leads to macromolecules leaks from the cell. This evidence elaborated the potential therapeutic efficacy of CPA 19 and provided a significant contribution to the discovery of antibacterial agents.

## Introduction

About 78 years ago, penicillin and other antibiotics were discovered as antimicrobial agents and introduced in various clinical treatments because of their positive influences in preventing bacterial infections (Paitan, 2018). However, Abraham and Chain (1988) reported the emergence of antimicrobial resistance.

Among these resistant bacterial strains, species of *E. coli* have emerged against the variety of antibiotic agents used in clinical practice, which not only damages the production of the animal industry, but also seriously affects the health of human beings. Therefore, there is an urgent need for the search of safe and effective approaches to overcome single as well as multidrug-resistant bacterial infections (Roth et al., 2019).

In recent years, more and more researchers have been devoted to the study of the inhibition of plant polyphenols because of their natural and broad antibacterial properties (Yun-Seok et al., 2010; Sidhu et al., 2014; Yang et al., 2020). Phenolic acids are major plant metabolites, which occur in all parts of the plant: shells, leaves, seeds, fruits, and wood parts (Efenberger et al., 2021). Previous studies have demonstrated that phenolic compounds possess a variety of biological functions including anti-carcinogenic, anti-inflammatory, and anti-oxidant properties. Moreover, some phenolic compounds have been proven to be effective in inhibiting various pathogenic bacteria such as *E. coli* (Cushnie and Lamb, 2005; Almajano et al., 2007; Martina, 2010), however, the antibacterial effect was not satisfactory, and their studies only assessed the level of the simple antibacterial mechanism. What is more, the components in plants are complex and diverse, it is impossible to determine effective specific antibacterial ingredients, and it is difficult to explain the mechanism of phenolic acids.

Therefore, in this study, five phenolic acids were combined with each other in order to obtain one of the most effective combinations against multidrug-resistant *E. coli*, which may provide a significant contribution to the field of antibiotic agent discovery.

## Materials and Methods

### Bacterial Strains

Seven strains of *E. coli* (E1, E2, E3, E4, E5, E6, and E7) from poultry were stored in Guangxi University (Nanning, China), which were confirmed by the analysis of 16S rDNA sequencing (Kim et al., 2010). The resistance genes of the seven *E. coli* strains are shown in Table 1 (identification by the 25 μL PCR reaction system).

**TABLE 1:** Resistance genes of the seven strains of *E. coli*.

Number of *E. coli*	Resistance genes
E1	*rmtB, tetA*, *tetM*, *bla*_OXA–1_, *bla*_CTX–m–u_, *bla*_CTX–m–1_, *sul1*, *sul2 sul3*, *floR*, *oqxA*
E2	*rmtB*, *tetA*, *tetM*, *bla*_OXA–1_, *bla*_CTX–m–u_, *bla*_CTX–m–1_, *sul1*, *sul2*, *oqxA*
E3	*armA*, *rmtA*, *rmtB*, *rmtC*, *tetA*, *bla*_*OXA–2*_, *bla*_CTX–m–1_, *qnrA, floR*, *oqxA*, *mcr-1*
E4	*rmtD*, *tetB*, *tetC*, *bla*_CTX–m–u_, *bla*_CTX–m–1_, *bla*_CTX–m–9_, *sul2*, *floR*, *mcr-1*
E5	*rmtD*, *tetA*, *tetM*, *bla*_TEM_, *bla*_OXA–10_, *bla*_CTX–m–u_, *bla*_CTX–m–9_, *qrnB*, *floR*, *oqxA*, *sul2*, *sul3*
E6	*rmtD*, *tetA*, *bla*_OXA–10_, *bla*_CTX–m–u_, *bla*_CTX–m–9_, *sul2*, *sul3*, *fosA3*, *mcr-1*
E7	*rmtA*, *rmtD*, *tetB*, *tetC*, *bla*_TEM_, *bla*_CTX–m–u_, *bla*_CTX–m–1_, *bla*_CTX–m–9_, *oqxA*, *sul1*, *sul2*

### Preparation of Compound Phenolic Acids

The five phenolic acids were dissolved with maximum solubility (salicylic acid, CAS: 69-72-7, 0.22 g/100 g H_2_O; protocatechuic acid, CAS: 99-50-3, 2 g/100 g H_2_O; gallic acid, GAS: 149-91-7, 1.14 g/100 g H_2_O; hydrocinnamic acid, GAS: 501-52-0, 0.5 g/100 g H_2_O; chlorogenic acid, GAS: 327-97-9, 4 g/100 g H_2_O), and 5% dimethyl sulfoxide (DMSO) was added to promote the solubility of phenolic acids. Finally, the phenolic acids were mixed with each other in equal volumes to obtain CPAs with different compositions.

### Determination of Minimum Inhibitory Concentrations

The microdilution method (Smekalova et al., 2016) was used to detect the MIC of each CPA on *E. coli*, and the results were statistically analyzed. Mueller-Hinton Broth (MHB) was used as the incubation medium. The CPAs (100 μL) were serially diluted in a 96-well plate by 100 μL of *E. coli* inoculum (approximately 1.5 × 10^5^ CFU/mL) for 12 times, and the final concentrations of CPAs were 50, 25, 12.5, 6.15, 3.125, 1.5625, 0.78125, 0.39065, 0.1953125, 0.09765625, 0.048828125, and 0.0244140625% of the initial concentration, respectively. After incubation at 37°C for 12–16 h, MICs were measured by visual inspection of the turbidity of broth in tubes. That is to say, if the test tube was still clear and transparent (un-cloudy) after incubation, the cells could not grow at that concentration and then the MIC value was obtained. Florfenicol (in China, florfenicol is an approved animal-specific antibiotic, but it is banned in laying hens) was used as a positive anti-*E. coli* control; MHB was used as a blank control.

### Tandem Mass Tag Quantitative Proteomics

#### Cultivation and Pre-treatment of *Escherichia coli*

*Escherichia coli* were cultured at 37°C until the logarithmic growth phase at 220 r/min. The optical density value (OD_600_) of the bacterial fluid was diluted to 0.6, and then evenly divided into two groups, each group was tested in triplicate. Group A was treated with CPA at the concentration of 1/2 MIC, while same amount of H_2_O was added in group B and kept as the control group. The mixture was incubated at 37°C for 60 min at 220 r/min, centrifuged for 15 min (4°C, 5000 r/min), and the supernatant was discarded. After repeated washing with RNA-free PBS three times, the samples were immediately frozen in liquid nitrogen, and then stored at −80°C for further use.

#### Protein Extraction

The samples were lysed by ultrasound using four times the volume of lysis buffer (8 M urea, 1% protease inhibitor). Following centrifugation at 12000 g for 10 min (to remove the cell debris), the supernatant was collected to determine the protein concentration using a BCA kit (A045-4-2, NanJing JianCheng Bioengineering Institute Co., Ltd., Nanjing, China).

#### Trypsin Digestion

For digestion, the protein solution was reduced with 5 mM of dithiothreitol for 30 min at 56 °C and alkylated with 11 mM of iodoacetamide for 15 min at room temperature in darkness. The protein sample was then diluted by adding 100 mM of TEAB to a urea concentration less than 2 M. Finally, trypsin was added at a 1:50 trypsin-to-protein mass ratio for the first digestion overnight and a 1:100 trypsin-to-protein mass ratio for a second 4-h digestion.

#### Tandem Mass Tag Labeling

After trypsin digestion, the peptide was desalted by a Strata X C18 SPE column (Phenomenex) and vacuum-dried. The peptide was reconstituted in 0.5 M of TEAB and processed according to the manufacturer’s protocol for the TMT kit. Briefly, one unit of TMT reagent was thawed and reconstituted in acetonitrile. The peptide mixtures were then incubated for 2 h at room temperature and pooled, desalted, and dried by vacuum centrifugation.

#### HPLC Fractionation

The tryptic peptides were fractionated into fractions by high pH reverse-phase HPLC using a Thermo Betasil C18 column (5 μm particles, 10 mm ID, 250 mm length). Briefly, peptides were first separated with a gradient of 8 to 32% acetonitrile (pH 9.0) over 60 min into 60 fractions. Then, the peptides were combined into six fractions and dried by vacuum centrifuging. LC-MS/MS Analysis.

The tryptic peptides were dissolved in 0.1% formic acid (solvent A), directly loaded onto a homemade reversed-phase analytical column (15-cm length, 75 μm i.d.). The gradient was comprised of an increase from 6 to 23% solvent B (0.1% formic acid in 98% acetonitrile) over 26 min, 23 to 35% in 8 min and climbing to 80% in 3 min then holding at 80% for the last 3 min, all at a constant flow rate of 400 nL/min on an EASY-nLC 1000 UPLC system. The peptides were subjected to NSI source followed by tandem mass spectrometry (MS/MS) in Q Exactive TM Plus (Thermo Fisher Scientific) coupled online to the UPLC. The electrospray voltage applied was 2.0 kV. The m/z scan range was 350 to 1800 for full scan, and intact peptides were detected in the Orbitrap at a resolution of 70,000. Peptides were then selected for MS/MS using the NCE setting at 28 and the fragments were detected in the Orbitrap at a resolution of 17,500. A data-dependent procedure that alternated between one MS scan followed by 20 MS/MS scans with 15.0 s dynamic exclusion was used. Automatic gain control (AGC) was set at 5E4. The fixed first mass was set as 100 m/z.

#### Database Search

The resulting MS/MS data were processed using the Maxquant search engine (v.1.5.2.8). Tandem mass spectra were searched against the human uniprot database concatenated with a reverse decoy database. Trypsin/P was specified as the cleavage enzyme allowing up to four missing cleavages. The mass tolerance for precursor ions was set as 20 ppm in the first search and 5 ppm in the main search, and the mass tolerance for fragment ions was set as 0.02 Da. Carbamidomethyl on Cys was specified as the fixed modification, and acetylation modification and oxidation on Met were specified as variable modifications. FDR was adjusted to < 1% and the minimum score for modified peptides was set > 40.

### Parallel Reaction Monitoring

Parallel reaction monitoring (PRM) mass spectrometric analysis was performed using tandem mass spectrometry (MS/MS) in Q ExactiveTM Plus (Thermo Fisher Scientific). The liquid chromatography parameters, electrospray voltage, scan range, and Orbitrap resolution were the same as the TMT methods. Automatic gain control (AGC) was set at 3 × 10^6^ for full MS and 1 × 10^5^ for MS/MS. The maximum IT was set at 20 ms for full MS and auto for MS/MS. The isolation window for MS/MS was set at 2.0 m/z. After the quantitative information was normalized by the heavy isotope-labeled peptide, a relative quantitative analysis (three biological replications) was performed on the target peptides.

### Fractional Inhibition Concentration Index of Antibiotics and Compound Phenolic Acids

Modified Lorian (2005) method was used to determine the interaction between antibiotics and CPAs. The CPA was diluted vertically, and the antibiotic was diluted horizontally in a 96-well plate. The following formula was used to calculate FICI:


$$\text{FICI}=\frac{\text{MIC}\,\left(\mathrm{antibiotic}\,\mathrm{in}\,\mathrm{combination}\right)}{\text{MIC}\,\left(\mathrm{antibiotic}\,\mathrm{alone}\right)}+\frac{\text{MIC}\,\left(\mathrm{CPA}\,\mathrm{in}\,\mathrm{combination}\right)}{\text{MIC}\,\left(\mathrm{CPA}\,\mathrm{alone}\right)}$$

The combined antimicrobial action of CPAs and antibiotics was determined according to FICI, the combined antibacterial effects were considered to be synergy, additivity, indifference, or oppositive when the FCIC was ≤ 0.5, > 0.5 to ≤ 1, > 1 to ≤ 2, and > 2, respectively (Kurek et al., 2012).

### Determination of Extracellular Soluble Protein

The CPA was added in a bacterial suspension (10^6^ CFU/mL) with a concentration of 1/2 MIC. The same volume of H_2_O was used as the control group. The mixture was cultured at 37°C with shaking at 220 r/min. Samples were collected separately from 0 to 18 h every hour. The determination of extracellular soluble protein was measured by a protein quantitative test kit (A045-2, NanJing JianCheng Bioengineering Institute Co., Ltd., Nanjing, China). Each group test was repeated three times.

### The Ultrastructure of Cells Was Observed by Transmission Electron Microscopy

The CPA was added to E6 at the logarithmic growth stage, and the final concentration was adjusted to 1/2 MIC, incubated at 37°C and 220 r/min for 4 h, and the same amount of H_2_O was used as a blank control. After centrifugation of the mixture (3 000 r/min, 15 min), the precipitation was collected and washed twice with PBS. A total of 1 mL of 3% glutaraldehyde was added to the bacterial plate and kept at 4°C overnight. The liquid was centrifuged (3 000 r/min, 30 min) and the supernatant was collected. After separation, the supernatant was washed with PBS three times, fixed with 1% osmium tetroxide, and kept at 4°C for 2 h. After this, the supernatant was uninterruptedly dehydrated using 30, 50, 75, 95, and 100% ethanol for dehydration. Epoxy resin was embedded at 60°C for 48 h and sliced (with 50-nm thickness) (Ultra 45°, Daitome). The samples were placed on a 400-mesh copper network and stained with 2.0% uranium dioxane acetate and lead citrate for 30 min. After washing with distilled water, the samples were dried at 37°C. Finally, the ultrastructural changes were observed under TEM (TECNAI G2 20 TWIN, FEI).

## Results

### Composition and Concentration of 31 Compound Phenolic Acids

A total of 31 combinations of CPAs were obtained; all components and their concentrations in each CPA are presented in Table 2. In our subsequent study, the initial concentration of each CPA was recorded as 1, which was not only convenient for recording, but also more intuitive to reflect the antibacterial effect. The specific calculation method of actual MIC values of each CPA in subsequent experiments is described in the second sentence of the next section.

**TABLE 2:** Composition and concentration of 31 combinations of CPAs.

Number	Composition and concentration of CPAs
1	Salicylic acid 1.1 mg/mL,protocatechuic acid 10 mg/mL
2	Salicylic acid 1.1 mg/mL, gallic acid 5.7 mg/mL
3	Salicylic acid 1.1 mg/mL,hydrocinnamic acid 2.5 mg/mL
4	Salicylic acid 1.1 mg/mL,chlorogenic acid 20 mg/mL
5	Protocatechuic acid 10 mg/mL,gallic acid 5.7 mg/mL
6	Protocatechuic acid 10 mg/mL,hydrocinnamic acid 2.5 mg/mL
7	Protocatechuic acid 10 mg/mL,chlorogenic acid 20 mg/mL
8	Gallic acid 5.7 mg/mL,hydrocinnamic acid 2.5 mg/mL
9	Gallic acid 5.7 mg/mL,chlorogenic acid 20 mg/mL
10	Hydrocinnamic acid 2.5 mg/mL,chlorogenic acid 20 mg/mL
11	Salicylic acid 0.73 mg/mL,protocatechuic acid 6.67 mg/mL,gallic acid 3.8 mg/mL
12	Salicylic acid 0.73 mg/mL,protocatechuic acid 6.67 mg/mL,hydrocinnamic acid 1.67 mg/mL
13	Salicylic acid 0.73 mg/mL,protocatechuic acid 6.67 mg/mL,chlorogenic acid 13.33 mg/mL
14	Salicylic acid 0.73 mg/mL,gallic acid 3.8 mg/mL,hydrocinnamic acid 1.67 mg/mL
15	Salicylic acid 0.73 mg/mL,gallic acid 3.8 mg/mL,chlorogenic acid 13.33 mg/mL
16	Salicylic acid 0.73 mg/mL, hydrocinnamic acid1.67 mg/mL, chlorogenic acid 13.33 mg/mL
17	Protocatechuic acid 6.67 mg/mL, gallic acid 3.8 mg/mL, hydrocinnamic acid 1.67 mg/mL
18	Protocatechuic acid 6.67 mg/mL, gallic acid 3.8 mg/mL, chlorogenic acid 13.33 mg/mL
19	Protocatechuic acid 6.67 mg/mL, hydrocinnamic acid 1.67 mg/mL, chlorogenic acid 13.33 mg/mL
20	Gallic acid 3.8 mg/mL, hydrocinnamic acid 1.67 mg/mL, chlorogenic acid 13.33 mg/mL
21	Salicylic acid 0.55 mg/mL, protocatechuic acid 5 mg/mL, gallic acid 2.85 mg/mL, hydrocinnamic acid 1.25 mg/mL
22	Salicylic acid 0.55 mg/mL, protocatechuic acid 5 mg/mL, gallic acid 2.85 mg/mL, chlorogenic acid 10 mg/mL
23	Salicylic acid 0.55 mg/mL, protocatechuic acid 5 mg/mL, hydrocinnamic acid 1.25 mg/mL, chlorogenic acid 10 mg/mL
24	Salicylic acid 0.55 mg/mL, gallic acid 2.85 mg/mL, hydrocinnamic acid 1.25 mg/mL, chlorogenic acid 10 mg/mL
25	Protocatechuic acid 5 mg/mL, gallic acid 2.85 mg/mL, hydrocinnamic acid 1.25 mg/mL, chlorogenic acid 10 mg/mL
26	Salicylic acid 0.44 mg/mL, protocatechuic acid 4 mg/mL, gallic acid 2.28 mg/mL, hydrocinnamic acid 1 mg/mL, chlorogenic acid 8 mg/mL
27	Salicylic acid 2.2 mg/mL
28	Protocatechuic acid 20 mg/mL
29	Gallic acid 11.4 mg/mL
30	Hydrocinnamic acid 5 mg/mL
31	Chlorogenic acid 40 mg/mL

### Determining the Compound Phenolic Acid With the Best Antibacterial Effect

Seven strains of *E. coli* were used to assess the antibacterial efficacy of all CPAs by the microdilution method. The results are shown in Table 3, taking E1 as an example, the MIC of CPA 1 on E1 was 25% of its initial concentration, at this time, the concentration of each component in CPA1 = 25%*1 (salicylic acid 1.1 mg/mL,protocatechuic acid 10 mg/mL), which meant 0.275 mg/mL of salicylic acid and 2.5 mg/mL of protocatechuic acid.

**TABLE 3:** The MICs of 31 combinations of CPAs against the 7 strains of *E. coli*.

Agents	MICs of *E. coli*						
	E1 (%)	E2 (%)	E3 (%)	E4 (%)	E5 (%)	E6 (%)	E7 (%)
CPA 1	25	25	25	25	25	25	25
CPA 2	25	25	25	25	25	25	50
CPA 3	50	50	50	50	50	50	25
CPA 4	25	25	25	25	25	25	25
CPA 5	25	25	25	25	25	25	25
CPA 6	12.5	12.5	12.5	12.5	12.5	25	25
CPA 7	25	25	12.5	25	25	25	25
CPA 8	25	25	25	25	25	25	25
CPA 9	25	25	25	25	25	25	25
CPA 10	12.5	25	25	25	25	25	25
CPA 11	25	25	25	25	25	25	25
CPA 12	25	25	25	25	25	25	25
CPA 13	25	25	25	25	25	25	25
CPA 14	25	25	25	25	25	25	25
CPA 15	25	25	25	25	25	25	25
CPA 16	25	25	25	25	25	25	25
CPA 17	25	25	25	12.5	25	25	25
CPA 18	25	25	25	25	25	25	25
CPA 19	12.5	12.5	12.5	12.5	12.5	12.5	12.5
CPA 20	25	25	25	25	25	25	25
CPA 21	25	25	25	25	25	25	25
CPA 22	25	25	25	25	25	25	25
CPA 23	12.5	25	25	12.5	12.5	25	25
CPA 24	25	25	25	25	25	25	25
CPA 25	25	25	25	12.5	25	25	12.5
CPA 26	25	25	25	25	25	25	12.5
CPA 27	>50	>50	>50	50	50	50	50
CPA 28	25	25	25	25	25	25	50
CPA 29	50	50	50	50	25	50	25
CPA 30	50	50	25	25	25	25	25
CPA 31	25	50	25	25	25	25	25
Florfenicol	1280	640	640	640	640	2560	640

*MICs were calculated with the initial concentration of CPA as 1, the unit of florfenicol was μg/mL.*

As shown in Table 3, all of the CPAs showed certain inhibitory effects on *E. coli*. The antibacterial effect of each combination was different due to different components and *E. coli* strains. However, compared to all other CPAs, CPA 19 showed better efficacy against all seven strains of *E. coli* and the MIC was reached at 12.5%. In other words, the concentrations of each component in the MIC (12.5% CPA 19) were 0.834 mg/mL (protocatechuic acid), 0.208 mg/mL (hydrocinnamic acid), and 1.67 mg/mL (chlorogenic acid). Therefore, CPA 19 was selected as a potential antimicrobial agent and its mechanisms were further explored against E6 *E. coli*.

### General Features of Proteome After Compound Phenolic Acid 19 Treatment

After treatment with a subinhibitory concentration of CPA 19, 2688 proteins were detected and identified, and 2560 were quantified. The detailed data (the protein score, coverage percentage, number of peptides matching individual proteins, and accession number assigned to each identified protein) of related proteins are shown in Supplementary Table 1. The MS proteomic results reported in this paper have been deposited in the OMIX, China National Center for Bioinformation/Beijing Institute of Genomics, Chinese Academy of Sciences, under accession number OMIX382 which is accessible at https://ngdc.cncb.ac.cn/omix.

### Differentially Expressed Proteins After Compound Phenolic Acid 19 Treatment

**FIGURE 1:**
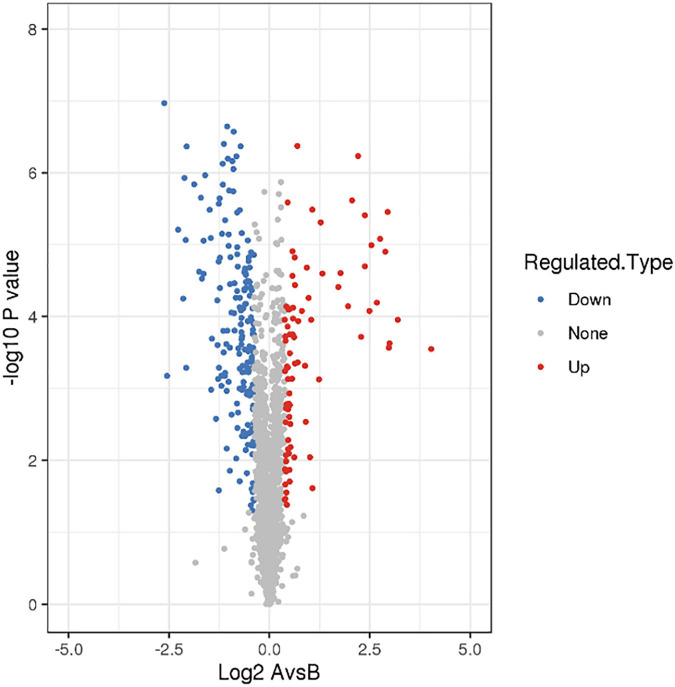
Screening of differentially expressed proteins.

There were 268 differentially expressed proteins after treatment with a subinhibitory concentration of CPA 19, including 84 upregulated proteins and 184 downregulated proteins, when the fold change was defined as greater than 1.3 or lower than 1/1.3, and *P* < 0.05 (Figure 1). Among them, there were 25/51 significantly up/downregulated proteins with more than two fold changes.

### Gene Ontology Functional Enrichment Analysis

**FIGURE 2:**
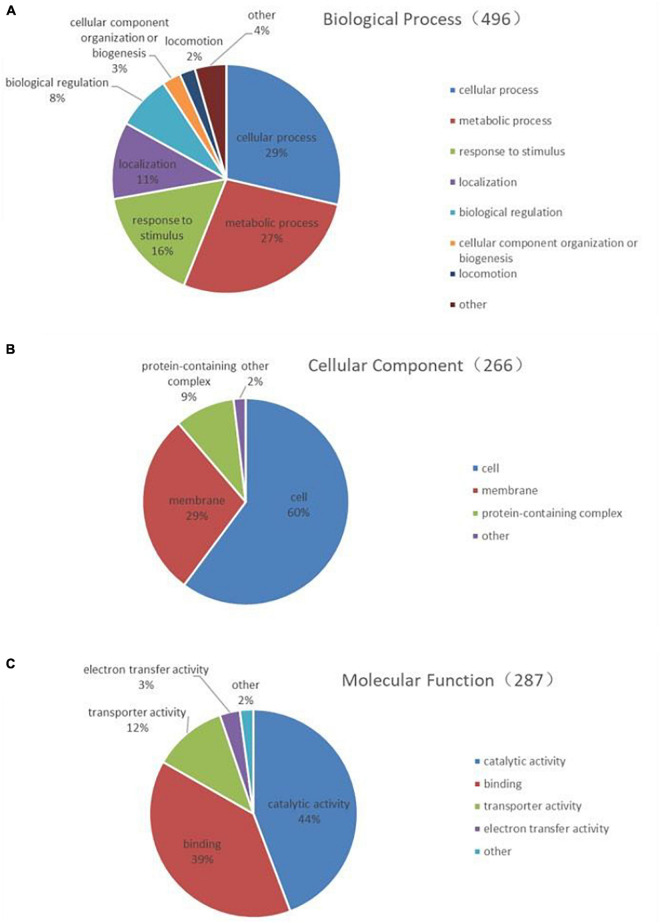
Functional classification of differentially expressed proteins **(A)** biological process, **(B)** cell composition, and **(C)** molecular function.

The 268 identified proteins were annotated by GO secondary classification, and the effect of the subinhibitory concentration of CPA 19 on multi-drug resistant *E. coli* was preliminarily analyzed (Supplementary Table 2). The distribution statistics of differentially expressed proteins in the three major categories of GO secondary annotations are shown in Figure 2. Biological process (Figure 2A) was mainly enriched with the proteins involved in cellular processes (29%), metabolic processes (27%), and response to stimulus (16%); in the classification of cell composition (Figure 2B), most of the differential proteins were distributed in cells (60%), membranes (29%), and the protein-containing complex (9%); in the molecular function classification (Figure 2C), there were mainly differentially expressed proteins in catalytic activity (44%), binding (39%), and transport activity (12%).

### Kyoto Encyclopedia of Genes and Genomes Pathway Enrichment Analysis

**FIGURE 3:**
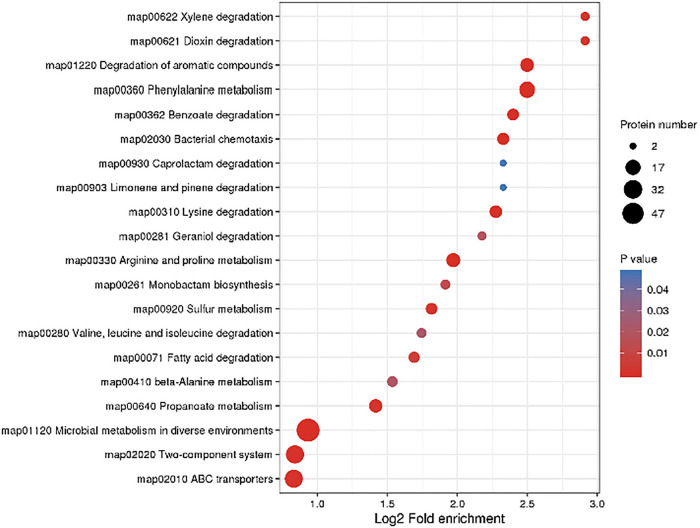
Significantly enriched KEGG pathways.

In order to further understand the effect of CPA 19 on multi-drug resistant *E. coli*, KEGG pathway enrichment analysis was performed on these 268 differentially expressed proteins (Supplementary Table 3). There were 11 significantly enriched (*P* < 0.05) KEGG upregulation pathways: Degradation of aromatic compounds, phenylalanine metabolism, sulfur metabolism, microbial metabolism in diverse environments, dioxin degradation, xylene degradation, monobactam biosynthesis, two-component system, selenocompound metabolism, benzoate degradation, and nitrotoluene degradation. And there were 18 significantly enriched (*P* < 0.05) KEGG downregulation pathways: Bacterial chemotaxis, arginine and proline metabolism, lysine metabolism, ABC transporter, propionate lipid metabolism, microbial metabolism in different environments, phenylalanine metabolism, beta-alanine metabolism, valine, leucine, and isoleucine degradation, fatty acid degradation, geraniol degradation, benzoic acid degradation, quorum sensing, limonene and pinene degradation, caprolactam degradation, tryptophan metabolism, two-component system, and methyl butyrate metabolism. The top 20 enriched pathways are shown in Figure 3.

**FIGURE 4:**
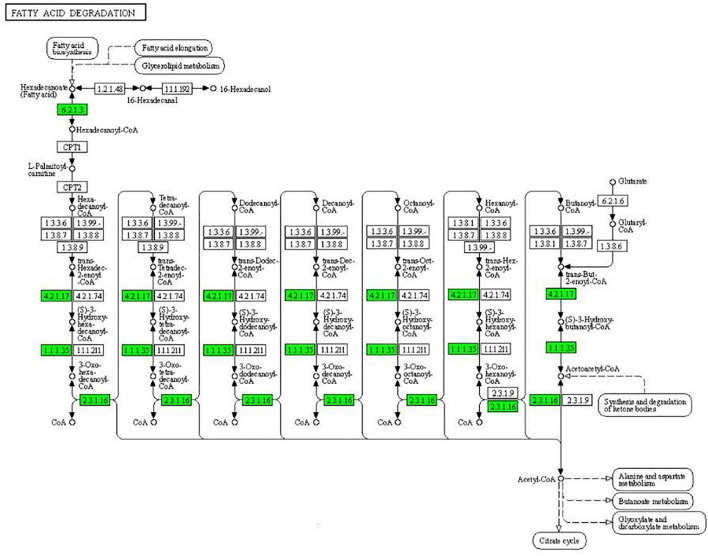
Significantly enriched KEGG pathway: Fatty acid degradation (from the KEGG database, green frames represent downregulation, and red frames represent upregulation).

**FIGURE 5:**
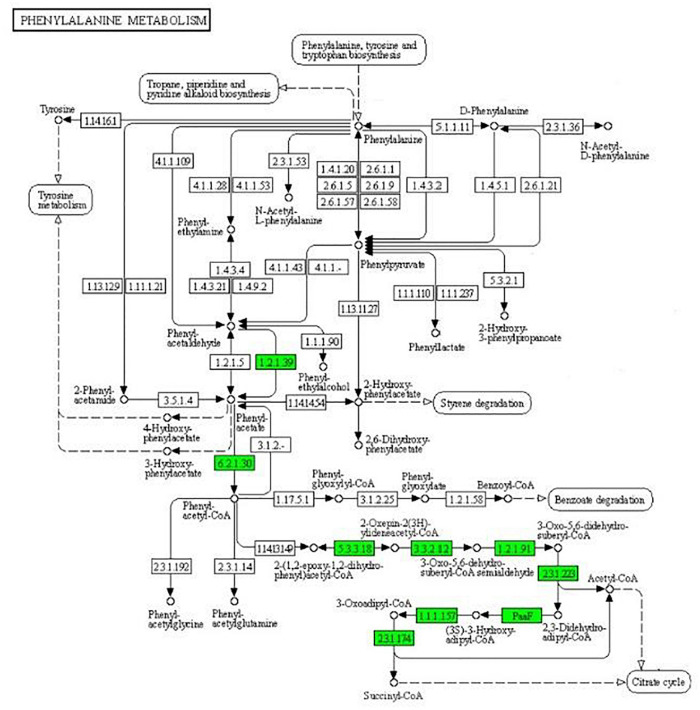
Significantly enriched KEGG pathway: Phenylalanine metabolism (from the KEGG database, green frames represent downregulation, and red frames represent upregulation).

**FIGURE 6:**
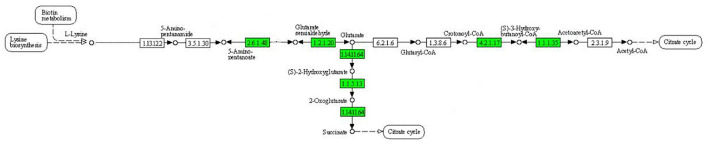
Significantly enriched KEGG pathway: Lysine metabolism (from the KEGG database, green frames represent downregulation, and red frames represent upregulation).

As can be seen from Figure 4, the expression of some important enzymes (long-chain acyl-CoA synthetase [EC:6.2.1.3], enoyl-CoA hydratase [EC:4.2.1.17], 3-hydroxyacyl-CoA dehydrogenase [EC:1.1.1.35], acetyl-CoA acyltransferase [EC:2.3.1.16]) regulating β oxidation were significantly downregulated in the fatty acid degradation pathway, indicating that the fatty acid degradation of multidrug-resistant *E. coli* was inhibited after treatment with the subinhibitory concentration of CPA 19, and leading to blocked generation of acetyl-CoA. Similarly, in the pathway of phenylalanine metabolism (Figure 5) and lysine metabolism (Figure 6), the metabolic process of phenylalanine and lysine were also stressed by CPA 19, which led to the obstruction of the conversion of acetyl-CoA.

**FIGURE 7:**
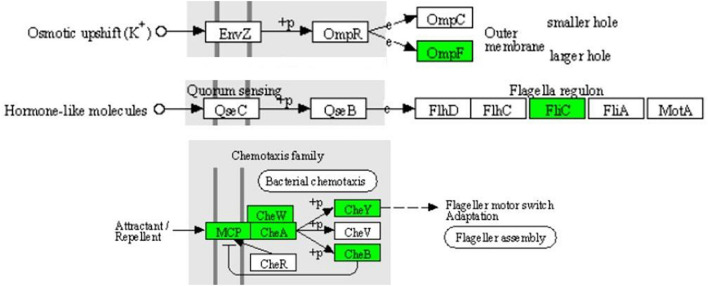
Significantly enriched KEGG pathway: Two-component system (from the KEGG database, green frames represent downregulation, and red frames represent upregulation).

**FIGURE 8:**
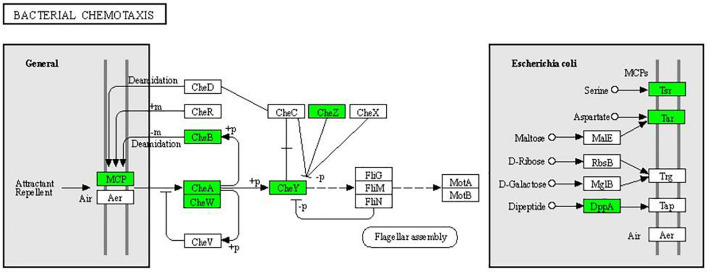
Significantly enriched KEGG pathway: Bacterial chemotaxis (from the KEGG database, green frames represent downregulation, and red frames represent upregulation).

In the two-component system (Figure 7), the expression of outer membrane pore protein F (OmpF) and flagellin (FliC) in the OmpR family, as well as methyl-accepting chemotaxis protein (MCP), purine-binding chemotaxis protein CheW (CheW), sensor kinase (CheA), chemotaxis protein CheY (CheY), and glutaminase (CheB) in the chemotactic family were significantly downregulated. MCP, CheW, CheA, CheY, and CheB were also significantly enriched in the bacterial chemotaxis pathway (Figure 8). At the same time, the expression of serine sensor receptor (Tsr) and aspartate sensor receptor (Tar) in the bacterial chemotaxis pathway were significantly downregulated.

### Parallel Reaction Monitoring Validation

We performed PRM verification on the significantly different expressed proteins in multi-drug resistant *E. coli* treated with the subinhibitory concentration of CPA 19. After evaluation of 50 differential proteins, 27 candidate proteins met the conditions, and 20 (10 upregulated, 10 downregulated) candidate proteins were selected for further verification. The results of PRM are shown in Table 4 and prove that all 20 target proteins were quantitative and consistent with the change trend of proteomics results, which supports the credibility and reliability of proteomics.

**TABLE 4:** PRM analysis of 20 candidate proteins.

Candidate proteins	A/B ratio	A/B *P* value	A/B ratio (TMT)
mhpB	331.32	0.000030	7.96
hcaB	206.09	0.000055	7.87
torA	209.67	0.000018	7.71
hcaD	82.12	0.000556	7.40
mhpC	339.84	0.000002	6.76
hcaE	202.34	0.000010	6.40
mhpD	190.85	0.000027	5.20
speF	1164.88	0.000001	4.87
mhpF	199.81	0.000028	3.42
mhpA	2.76	0.001827	2.36
CadA	0.31	0.000213	0.41
DUF1471 domain-containing protein	0.14	0.000012	0.37
yagU	0.15	0.000095	0.36
speC	0.09	0.000040	0.36
pdhR_2	0.14	0.000045	0.33
yeaW	0.10	0.000002	0.32
prpB	0.15	0.000001	0.31
cheM	0.13	0.000015	0.30
puuD	0.15	0.000031	0.27
tas	0.16	0.000005	0.23

### The Results of the Interaction Between Antibiotics and Compound Phenolic Acid 19

It can be seen from Table 5 that the FICI of CPA 19 and ceftiofur sodium was 1.5, which meant the interaction was indifferent, while the interaction of CPA 19 and azithromycin was oppositive. In addition, when combined with ceftriaxone sodium, amoxicillin, fosfomycin, sulfamonomethoxine, gatifloxacin, lincomycin, florfenicol, cefotaxime sodium, and rifampicin, additive effects were expressed. The MICs of several antibiotics (ceftriaxone sodium, amoxicillin, fosfomycin, sulfamonomethoxine, gatifloxacin, lincomycin, florfenicol, cefotaxime sodium, and rifampicin) after the combination with CPA 19 were all reduced by four times or more, which meant the antibacterial activities in vitro were significantly enhanced.

**TABLE 5:** The FICI of CPA 19 with antibiotics (E6).

Name of antibiotics	MIC (μg/mL)	Drug combination MIC		FICI	Effect
		Antibiotics (μg/mL)	CPA 19 (%)		
Ceftriaxone sodium	1600	<0.78125	6.25	0.5004	A
Amoxicillin	1600	<0.78125	6.25	0.5004	A
Fosfomycin	>3200	200	6.25	0.5625	A
Sulfamonomethoxine	>3200	<0.78125	6.25	0.5002	A
Gatifloxacin	100	6.25	6.25	0.5625	A
Lincomycin	3200	<0.78125	6.25	0.5002	A
Ceftiofur sodium	400	400	6.25	1.5	I
Florfenicol	2560	640	6.25	0.75	A
Azithromycin	100	200	6.25	2.5	O
Cefotaxime sodium	50	6.25	6.25	0.625	A
Rifampicin	1600	<0.78125	6.25	0.5004	A

*MICs were calculated with the initial concentration of CPA as 1. S, synergy; A, additivity; I, indifference; O, oppositive.*

### Effect of Compound Phenolic Acid 19 on Cell Membrane

**FIGURE 9:**
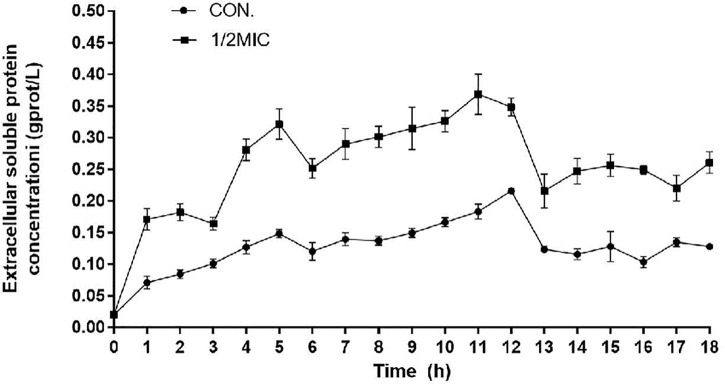
Variation diagram of extracellular soluble protein concentrations.

As shown in Figure 9, the concentration of soluble protein in the culture medium of the 1/2 MIC-treated group was significantly higher than in the culture medium of control group for the previous 12 h. During 13–18 h of incubation, the extracellular soluble protein tended to decline, which might be due to the antibacterial components gradually losing their effects with the lengthening of time.

### Effect of Compound Phenolic Acid 19 on Morphology

**FIGURE 10:**
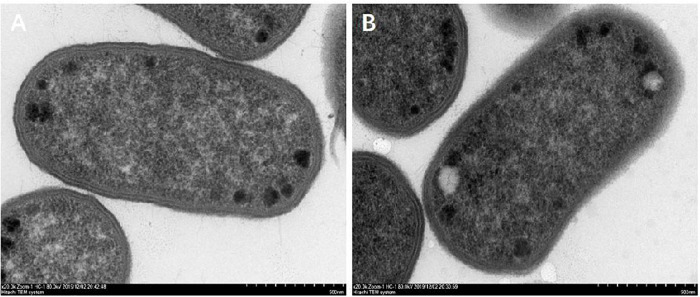
Morphological changes after CPA treatment. **(A)** CON and **(B)** 1/2 MIC.

The morphological changes of *E. coli* cells are shown in Figure 10. The cell wall of the control group was compact, complete, smooth with uniform cytoplasm, and flagellated (Figure 10A). After the treatment of CPA 19 with 1/2 MIC, significant changes were observed in the bacterial cell wall, the surface area of the cell wall became rough and the boundary between the cell wall and cell membrane became fuzzy, in addition, there was no flagellum on the surface of the bacterial cell (Figure 10B).

## Discussion

Phenolic acids, a major component in plants, have been shown to have antioxidant and antimicrobial activity against Gram-positive and Gram-negative bacteria (Shen et al., 2020). In addition, the order of antibacterial activity of the active ingredients in plants is: phenol > aldehyde > ketone > alcohol > ether > hydrocarbon (Nguyen et al., 2018). In this study, 31 CPAs of five phenolic acids were used to explore new and natural antimicrobial agents against multi-resistant *E. coli*. Our results of the MICs revealed the antibacterial effects of each CPA. However, the inhibitory effect of each CPA was different against all examined strains of *E. coli*. A previous study reported that the better therapeutic action of phenolic acids can be achieved from the synergistic effect of complex plant phenolic acid mixtures rather than from individual phenolic acid compounds (Coman and Vodnar, 2020). Moreover, the composition of plants is too complex and diverse to explain its antibacterial mechanism, which greatly limits its further development in clinical applications. Therefore, five phenolic acids were selected to explore the new antimicrobial agents, which could not only make the composition clear, but also retain the better antibacterial effect of plant phenolic acid mixtures when compared with single phenolic acids. Compared to all other CPAs, CPA 19 showed the best antibacterial efficacy. These findings implied that CPAs are better than individual compounds, and the best antibacterial effect of combination 19 not only elaborated the synergism of phenolic acids, but also represented its potential role against all examined multidrug-resistant *E. coli*.

The tricarboxylic acid cycle (TAC) is the final metabolic pathway through which the three nutrients (carbohydrates, lipids, and amino acids) provide and interconvert energy. These three nutrients can be metabolized to produce acetyl-CoA and then participate in TAC (Fan et al., 2021). Lipids are degraded by fatty acid degradation to generate acetyl-CoA followed by β-oxidation in the cytoplasm by activation of prokaryotic fatty acids. β-oxidation is a cyclic reaction of dehydrogenation, hydration, dehydrogenation, and thiolysis until the acyl-CoA is completely cleaved to acetyl-CoA (Yunqiao Yang et al., 2020). After CPA 19 treatment, the expression of some key enzymes in the β-oxidation process was downregulated, and the downregulation of these key proteins resulted in the decrease of acetyl-CoA. In the meanwhile, the conversion process of acetyl-CoA in the phenylalanine metabolism pathway and lysine metabolism pathway also could be impeded. These findings suggested that the CPA treatment may inhibit *E. coli* by perturbing the production of acetyl-CoA, and thus interrupt the material conversion and energy metabolism of *E. coli*.

Proteomic technology was used to analyze the differentially expressed proteins between multidrug-resistant *E. coli* and ATCC25922, which found that resistance of *E. coli* is associated with bacterial chemotaxis. Compared with ACCC25922, the expressions of Tsr, Tar, CheA, and CheW in resistant *E. coli* were significantly upregulated, indicating that these proteins are involved in the resistance of multidrug-resistant *E. coli* (Xia et al., 2020). What is more, Tsr, CheA, CheW, and CheR have also been shown to be related to the resistance of *Bacillus* and *Aeromonas hydrophila* (Wada et al., 2016). In our experiment, after treatment with CPA 19, the expression of Tsr, Tar, CheA, and CheW was significantly downregulated, suggesting that treatment with CPA 19 may restore the sensitivity of multidrug-resistant *E. coli* to antibiotics.

Combination with antibiotics as a synergist is also an important direction for the development of new antimicrobial agents. Yan (2020) reported that the combination of honokiol and polymyxin showed synergistic effects on *Enterobacteriaceae*. Further investigation has shown that honokiol could directly bind to the active region of MCR-1 and had competition for the specific binding of MCR-1 to the substrate, thus restoring the sensitivity of MCR-1-positive strains to polymyxin. In our results, CPA 19 also showed additive effects when combined with ceftriaxone sodium, amoxicillin, and cefotaxime sodium, which suggested that treatment with CPA 19 can indeed enhance the sensitivity of multidrug-resistant *E. coli* to antibiotics.

There have been studies that reported that bacterial chemotaxis had a regulatory effect on bacterial flagella, especially CheA and CheY (Yuhai et al., 2008; Yuan et al., 2012). In this study, not only were CheA and CheY downregulated, but also FliC. The downregulation of these proteins was also corroborated by flagella shedding on the surface of *E. coli* treated with phenolic acid 19 which was observed via TEM.

Destruction of the cell membrane is the major target of various antimicrobial agents, including phenolic acids (Fang et al., 2018). The antibacterial effect of phenolic acids may be due to the mechanism of the phenol-membrane interaction, isolation of metal ions, and inhibition of enzyme activity (Mirjana et al., 2005; Kemperman et al., 2010; Wang et al., 2010). As an important component of the cell membrane, OmpF crosses the phospholipid bilayer which is closely related to bacterial resistance of *E. coli* and the entry and exit of intracellular molecules (Jo-Anne and Elizabeth, 1997). In the two-component system, the expression of OmpF was significantly downregulated by the treatment of CPA 19, suggesting that the integrity of the membrane of the *E. coli* may be damaged. Macromolecular proteins originally present in the cell membrane and cytoplasm, which can be used to evaluate the integrity of the cell membrane (Vivek et al., 2013; Yunbin et al., 2016). The determination of extracellular soluble protein after CPA 19 treatment confirmed the downregulation of OmpF and cell membrane damage. These findings were in great agreement with Chenjie (Chenjie et al., 2015), they reported that the treatment of lactic acid perforated cell membranes, leaked intracellular material, and changed the morphology of *E. coli*. Phenolic acids can alter the permeability of cell membranes, damage the integrity of cells, not only causing the leakage of macromolecular substances, but also making it easier for harmful substances outside the cell to enter, which will eventually lead to cell death (Moreno et al., 2006). It could be seen from the results of this study that the damage of CPA 19 to the cell membrane might be the reason for the increased sensitivity of multidrug-resistant *E. coli* to antibiotics, and the antibiotics found it easier to enter the cell interior to play a role. In short, the cell membrane was also one of the targets of CPA 19.

## Conclusion

Compound phenolic acid 19, composed of protocatechuic acid, hydrocinnamic acid, and chlorogenic acid, had a good antibacterial effect against multi-drug resistant *E. coli*. When combined with antibiotics (ceftriaxone sodium, amoxicillin, fosfomycin, sulfamonomethoxine, gatifloxacin, lincomycin, florfenicol, cefotaxime sodium, and rifampicin), CPA 19 increased the susceptibility of *E. coli* to antibiotics. CPA 19 could shed the flagellum of multidrug-resistant *E. coli*, break the structure of the cell membrane, and cause the macromolecules to leak out from the cell. These data suggested that the natural compounds represented by CPA 19 in this study can be further developed as novel antimicrobial agents, and the use of CPA 19 in combination with antibiotics can be further explored for expression patterns of proteins involved in *E. coli* resistance.

## Data Availability Statement

The datasets presented in this study can be found in online repositories. The names of the repository/repositories and accession number(s) can be found in the article/Supplementary Material.

## Author Contributions

HS designed the work. All authors reviewed and approved the final manuscript.

## Conflict of Interest

The authors declare that the research was conducted in the absence of any commercial or financial relationships that could be construed as a potential conflict of interest.

## Publisher’s Note

All claims expressed in this article are solely those of the authors and do not necessarily represent those of their affiliated organizations, or those of the publisher, the editors and the reviewers. Any product that may be evaluated in this article, or claim that may be made by its manufacturer, is not guaranteed or endorsed by the publisher.
